# Whole-Genome Methylation Analysis Reveals Epigenetic Variation in Cloned and Donor Pigs

**DOI:** 10.3389/fgene.2020.00023

**Published:** 2020-02-20

**Authors:** Mengfen Wang, Shuaifei Feng, Guanjun Ma, Yiliang Miao, Bo Zuo, Jinxue Ruan, Shuhong Zhao, Haiyan Wang, Xiaoyong Du, Xiangdong Liu

**Affiliations:** ^1^Key Laboratory of Swine Genetics and Breeding of Ministry of Agriculture & Key Laboratory of Agriculture Animal Genetics, Breeding and Reproduction of Ministry of Education, College of Animal Science, Huazhong Agricultural University, Wuhan, China; ^2^Key Lab of Swine Healthy Breeding of Ministry of Agriculture and Rural Affairs, Guangxi Yangxiang Co., Ltd., Guigang, China; ^3^Hubei Key Laboratory of Agricultural Bioinformatics, College of Informatics, Huazhong Agricultural University, Wuhan, China

**Keywords:** DNA methylation, gene, immunity, methylation differential, pig, reproduction, somatic cloning

## Abstract

Somatic cloning has had a significant impact on the life sciences and is important in a variety of processes, including medical research and animal production. However, the application of somatic cloning has been limited due to its low success rate. Therefore, potential epigenetic variations between cloned and donor animals are still unclear. DNA methylation, one of the factors which is responsible for phenotypic differences in animals, is a commonly researched topic in epigenetic studies of mammals. To investigate the epigenetic variations between cloned and donor animals, we selected blood and ear fibroblasts of a donor pig and a cloned pig to perform whole-genome bisulfite sequencing (WGBS). A total of 215 and 707 differential methylation genes (DMGs) were identified in blood and ear fibroblasts, respectively. Functional annotation revealed that DMGs are enriched in many pathways, including T/B or natural killer (NK) cell differentiation, oocyte maturation, embryonic development, and reproductive hormone secretion. Moreover, 22 DMGs in the blood and 75 in the ear were associated with immune responses (e.g., *CD244*, *CDK6*, *CD5*, *CD2*, *CD83*, and *CDC7*). We also found that 18 DMGs in blood and 53 in ear fibroblasts were involved in reproduction. Understanding the expression patterns of DMGs, especially in relation to immune responses and reproduction, will reveal insights that will aid the advancement of future somatic cloning techniques in swine.

## Introduction

Somatic cell nuclear transplantation (SCNT) technology has been widely studied in basic research in medical science and livestock production. SCNT has been used to successfully clone different mammals (Li et al., 2006; Ogura et al., 2013). SCNT can produce genetically identical animals, and the production performance of the cloned animal can be similar to that of the donor. Consequently, SCNT may be an effective method for the production of indigenous target animals for use in epigenetic research (Adachi et al., 2014; Keefer, 2015; Lee et al., 2018). However, somatic cell cloning has a low efficiency success rate, which may be affected by somatic cell type and oocyte activation status (Akagi et al., 2014). Although several methods have been used to improve cloning efficiency, abnormal phenotypes that occur due to cloning continue to limit the utilization of this technology in industry. Somatic cell cloning inefficiency and phenotypic abnormalities are probably related to nuclear reprogramming, which may lead to a shortened life span for the cloned animals as well as diseases including, but not limited to severe pneumonia and thymic hypertrophy (Rideout et al., 2001; Ogura et al., 2002; Shimozawa et al., 2006). All of the aforementioned phenomena suggest that cloning may lead to low immunity in the cloned animals. However, several studies have reported that cloned animals had similar characteristics to the donor animals. For example, it has been reported that cloned Landrace and Jinhua sows grew normally and had similar carcass characteristics and reproductive performance compared to their offering and/or control group (Shibata et al., 2006; Adachi et al., 2014). Therefore, cloning holds great promise as a tool to produce elite breeding livestock. However, there is a lack of comparative studies on the reproductive performance and methylation of cloned and donor pigs which means that phenotypic and epigenetic differences between cloned and donor pigs are as yet unknown.

DNA methylation, the most common epigenetic modification in eukaryotes, alters the activity of non-coding elements such as introns, resulting in changes in the mechanism of action of DNA-proteins, and subsequent alterations in mammalian phenotypes (Jones and Takai, 2001). Mammalian DNA methylation mainly occurs in 5-methylcytosine and is associated with gene silencing (Li and Zhang, 2014), which is essential for normal mammalian development and normal biological phenotypes (Auclair and Weber, 2012; Bogdanovic and Gomez-Skarmeta, 2014). Similarly, epigenetic differences in monozygotic twins are an important cause of autoimmune diseases such as rheumatoid arthritis and type I diabetes in one of the twins (Hannon et al., 2018). A previous study has shown that, sows with different levels of DNA methylation have a difference litter size and different methylation gene mRNA expression levels (Hwang et al., 2017b). Sows cloned using ear fibroblasts display DNA methylation variation during the cloning process, and this variation leads to a short lifespan and organ dysplasia of cloned sows from the same pig (Shen et al., 2012). To investigate whether cloning will lead to a variation in DNA methylation, and subsequent changes in immune responses and reproductive performance in pigs. This work aims to describe the characterization of these epigenetic changes in the examined tissues of a cloned sow, in order to provide some insights for genetic studies of cloning.

## Materials and Methods

### Animals and Sample Collection

We cloned a normal female piglet from a fourth-parity Danish Yorkshire sow with an average litter size ≥18. Next, we collected the piglet's ear fibroblasts on the day of delivery for SCNT. Fibroblasts were separated by trypsin and collagenase for cell culture. The surrogate sow was normal, four-litter, gentle, and manageable. This sow also had a well-developed reproductive system, with two consecutive estrous periods. Its previous two litters consisted of 14 live piglets. A cloned female piglet was successfully cloned from the cultured piglet somatic cell population according to the nuclear transfer procedure of the cloned pig (Polejaeva et al., 2000). The cloned female pig and the donor female piglet were referred to as CP and DP, respectively. The CP and DP were both raised under the same environmental conditions and management. Blood is an important tissue which is associated with disease, immunity, and hormones. The methylation patterns of blood from the CP and DP were analyzed and found to be associated with litter size (Zhang et al., 2017a; Hwang et al., 2017b). Both blood and ear fibroblast samples from the CP and DP were collected for subsequent WGBS studies. Hereafter, blood and ear samples from the DP will be referred to as DP-blood and DP-ear, respectively. The same nomenclature will also be used for the CP. Both animals were raised in a fully enclosed barn with negative pressure ventilation, water curtain cooling, and a fully slatted floor. Both the DP and CP were fed alongside normal pregnant sows with a restricted feed regime and sufficient water during their pregnancy. All animal experiments were approved by the Scientific Ethic Committee of Huazhong Agricultural University (HZAUCA-2016-008).

### DNA Extraction, Bisulfite Treatment, and cDNA Preparation

Ear vein blood (3 ml) and ear fibroblast (2 g) samples were collected from the 594-day-old DP and CP for DNA extraction and subsequent WGBS studies. Genomic DNA was extracted using the TIANamp genomic DNA kit (TIANamp Technologies, Beijing, China) and bisulfite conversion was carried out using the EZ DNA Methylation Direct Kit (Zymo Research Corporation, Orange County, CA, USA). DNA quality and quantity were determined by NanoDrop (NanoDrop Technologies, Wilmington, DE, USA).

### WGBS Library Preparation and Data Quality Control

Four samples (DP-blood, DP-ear, CP-blood, and CP-ear) from the two pigs were used for WGBS sequencing. After a quality control test for the library preparation, these DNA samples were fragmented by Scientz18-A interrupter (SCIENTZ, Ningbo, China). The fragments were then end-repaired, plus A at the 3′ end, and ligated with adapters. Fragments of 400–500 base pairs (bp) were selected by agarose gel electrophoresis, then treated with bisulfite and subjected to PCR amplification to form the sequencing library. The sequencing library which passed a quality control test was sequenced using Illumina HiSeq Xten (Biomarker Technologies, Beijing, China). The sequencing reads were converted into FASTQ files by Illumina Casava1.8. To ensure the quality of the sequencing reads, we used FastQC software with the following criteria: (1) the removal of duplicate reads due to PCR amplification, (2) reads have adapters, (3) reads with more than 10% N content or more than 50% low quality bases. The raw sequencing data were stored in the National Centre for Biotechnology Information (NCBI) public database of Sequence Read Archive (SRA) (data number SUB5307644).

### Mapping Reads to the Reference Genome

After bisulfite treatment and PCR amplification, the unmethylated cytosines (C) from the genome were converted to thymine (T), and the methylated C remained unchanged. All of the C T conversions in the coding strand and guanine adenine (G A) conversions in the noncoding strand of the genome were compared with the reference genome (Sus_scrofa11.1) using Bowtie2, and the unique matches were subsequently analyzed. The filtered clean reads that aligned with the same region of the genome were used to summarize the sequencing depth and coverage (Krueger and Andrews, 2011). The percentage of methylated clean reads compared to the total number of clean reads in the lambda genome was calculated as the conversion rate of bisulfite using the Bismark software. The Bismark_methylation_extractor was used to identify and calculate the distribution of different types (CG, CHG, and CHH) of methylated C reads (Krueger and Andrews, 2011). Considering the sample size, we mapped the clean reads to the reference genome four times and retained only the reads that mapped to the same position of the reference genome four times for subsequent bioinformatic analysis. A binomial distribution test was performed to identify 5mC for each C site, and then the *P* value of binomial distribution test was subjected to false discovery rate (FDR) correction using the Benjamini & Hochberg method in the p.adjust package of the R software. The C site with coverage > 4X and an FDR < 0.05 was considered to be a true methylation site (Zhang et al., 2017b; Furci et al., 2019).

### Estimating 5mC Methylation Levels and Identifying Differential Methylation Regions (DMRs)

The methylation level of the C site with methylation frequencies are higher than the expected background in the bisulfite conversion reaction and sequencing errors in the binomial test detection were estimated. The methylation level was defined as the ratio between the number of reads of a methylated C to the total number of reads of that site (Schultz et al., 2012). We used the Kolmogorov–Smirnov test to determine the significance of the methylation differences between the CP and the DP, *p* < 0.05 was considered to be statistically significant. We also calculated the correlation of methylation levels by using the Pearson correlation coefficient of the samples. The regions with different methylation sites greater than 3, and in which the difference in methylation levels was no less than 0.2 (0.3 for CG type) with p value from Fisher's exact test of less than 0.05.) were used for DMR analysis with MOABS (Sun et al., 2014).

### Gene Ontology and Pathway Analysis

In order to identify genes associated with DMRs and to understand their functions, we used a homemade perl script (Enrich_analysis.pl) to perform a Kyoto Encyclopedia of Genes and Genomes (KEGG) pathway enrichment analysis. We also used topGO to perform gene function enrichment analysis *via* Gene Ontology (GO). GO terms and KEGG pathways with corrected *p*-value <0.05 were defined as enriched clusters. The genes that were located within the DMRs or closest to the DMRs of the intergenic region were detected and defined as differential methylation genes (DMGs) by ChIPseeker on the R package. All the DMGs underwent functional analysis using GO and KEGG.

## Results

### Reproductive Phenotype of the CP and DP

Under the same feeding conditions, the litter size of first three parities of the CP was 8, 12, and 21. The litter size of the first three parities of the DP was 12, 20, and 14. The total litter size of the CP was 4 piglets less than that of the DP in the first two parities and one piglet less in the first three parities (**Table 1**).

**Table 1:** Reproduction traits of the two pigs.

	Parity	Calving Age	Total Number Born	Number Born Alive	Stillbirth	Mummy
Donor pig (DP)	1	363	12	12	0	0
	2		20	20	0	0
	3		14	14	0	0
Cloned pig (CP)	1	414	8	8	0	0
	2		16	16	0	0
	3		21	20	1	0

Calving Age, the age (days) at the calving day. Total Number Born, the number of piglets produced by sows, including living piglets, stillbirths and mummies. Number Born Alive, living piglets at birth. Stillbirth, piglets born dead at birth. Mummy, dry corpse at birth.

### DNA Methylation Mapping and Patterns

Genome bisulfite sequencing results are shown in **Table 2**. A total of 366.43 GB of clean sequence data was obtained for the four samples. Appropriately 91.61 GB of clean data was obtained for each sample, with an average sequencing depth of equal to or greater than 25X. Methylation conversion with more than 87.82% of reads have >10X coverage and the quality control analysis showed that Q30 of the sequence data were more than 90.72%. In the mapping step, 74.93% of reads were aligned to the reference genome. These results suggested that our sequencing data was of high quality, which is beneficial for subsequent bioinformatic analyses.

**Table 2:** Whole genome DNA bisulfite sequencing data.

Pigs	Samples	Average depth(X)	Clean Base(GB)	Clean Reads	Mapped(%)	Conversion rate(%)	Total_ mc(%)
Donor pig	DP-blood	25	82.46	275101143	75.61	99.73	94.52
	DP-ear	29	94.26	314574836	74.93	99.64	94.62
Cloned pig	CP-blood	28	93.19	310889264	75.08	99.72	95.15
	CP-ear	29	96.51	322024573	74.97	99.65	95.41

Clean Bases (GB), number of bases after filtering. Clean Reads, the number of reads after filtering. Mapped (%), the percentage of Clean Reads mapped to the reference genome of all Clean Reads. Conversion rate (%), the percentage of Clean Reads aligned to lambda DNA and supported for methylation after treatment with bisulfite as a percentage of the total number of Clean Reads to lambda DNA. Total_mc (%), the number of clean reads matched to the reference genome relative to the amount of methylated cytosine within the clean reads matched to the reference genome.

**Figure 1:**
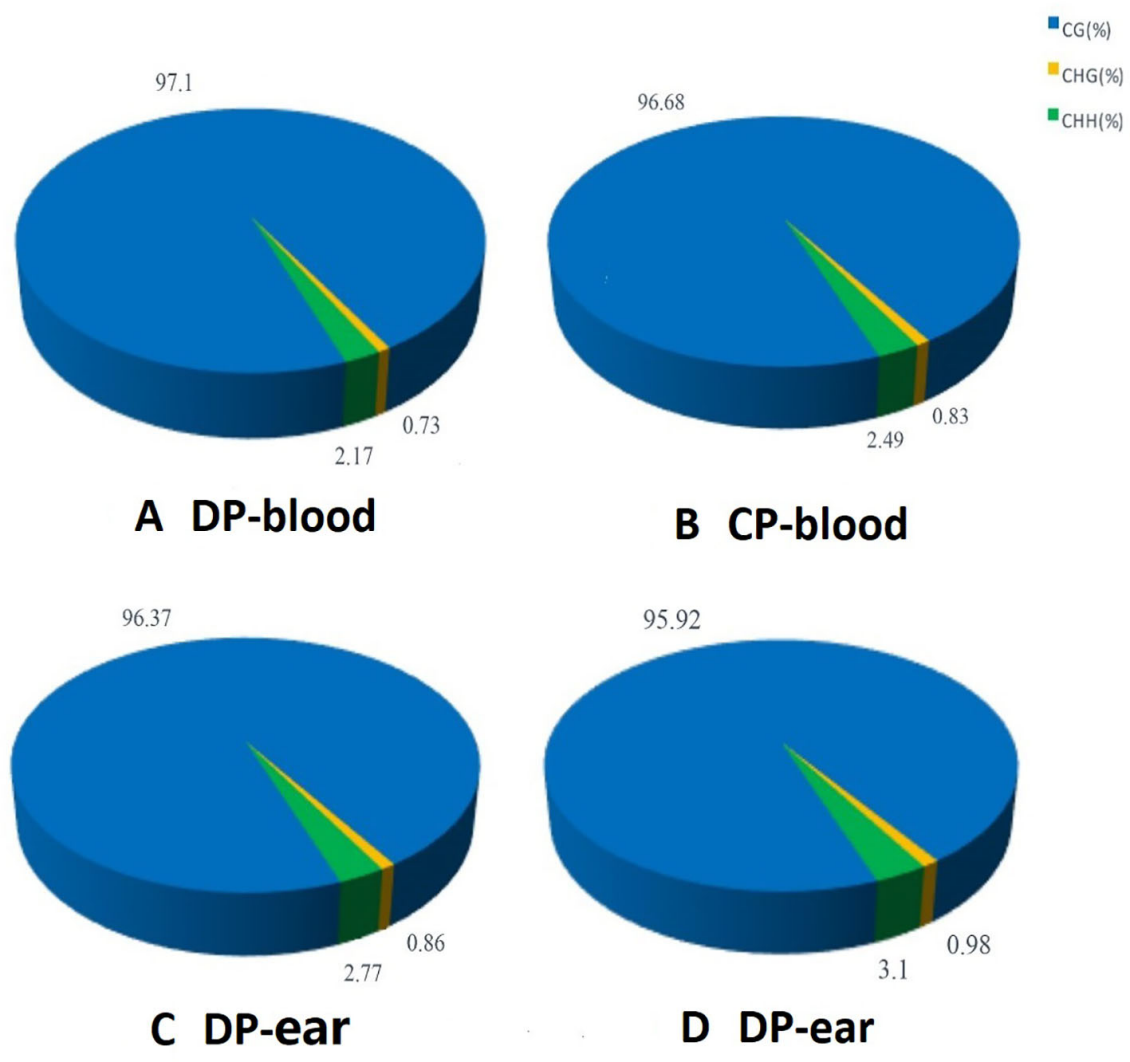
The average ratio of DNA methylation types in the genome of donor and cloned pigs. Panels **(A–D)** are the methylation patterns of DP-blood, CP-blood, DP-ear and CP-ear, respectively. The color of the pie chart indicates the type of methylation, and the number represents the proportion of the methylation type.

Three methylation patterns of CG, CHG, and CHH (H = A, T, or C) were obtained, and the methylated contexts and proportions of the four samples were similar. Methylated C in the blood sample of the DP was composed of 97.10% CG, 0.73% CHG, and 2.17% CHH compared to 96.68% CG, 0.83% CHG, and 2.49% CHH in the CP. Methylated C from ear tissue samples was composed of 96.37% CG, 0.86% CHG, and 2.77% CHH in the DP, and 95.92% CHG, 0.98% CHG, and 3.1% CHH in the CP (**Figure 1**). The majority of the methylation was CG, indicating that this type of methylation is dominant in this breed of pigs.

### Methylation of Different C Base Types

**Figure 2:**
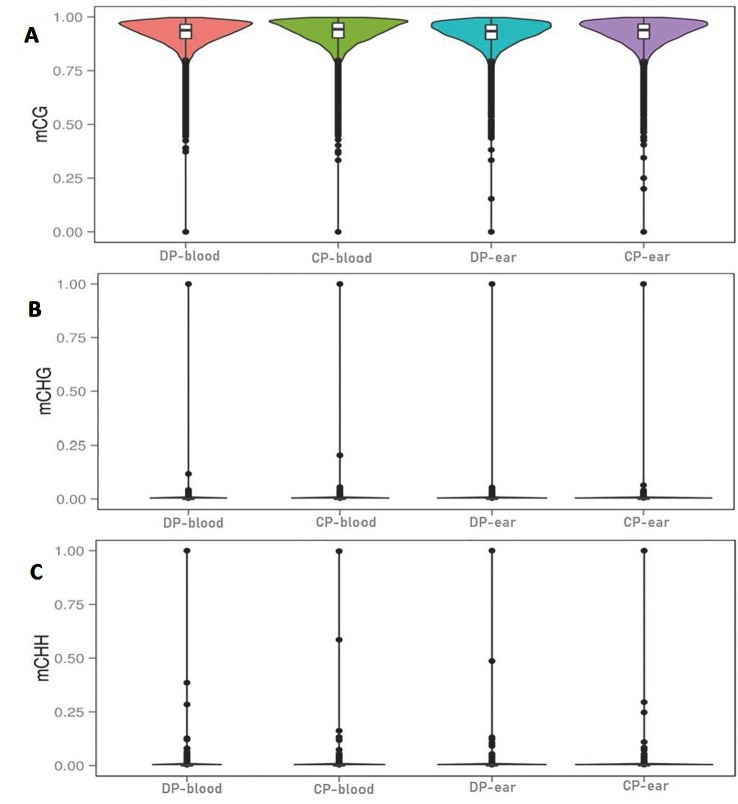
Violin plot for the overall distribution of methylation levels for different methylation types. Panels **(A–C)** are the context of CG, CHG, and CHH. H = A, C, or T. DP-blood, CP-blood, DP-ear, and CP-ear were the sample name. The abscissa represents the different samples, the ordinate represents the level of methylation of the samples; the points represented the methylation levels and cross-sectional area indicated the density of the methylation level; the boxplot shows the methylation levels in each violin.

**Figure 3:**
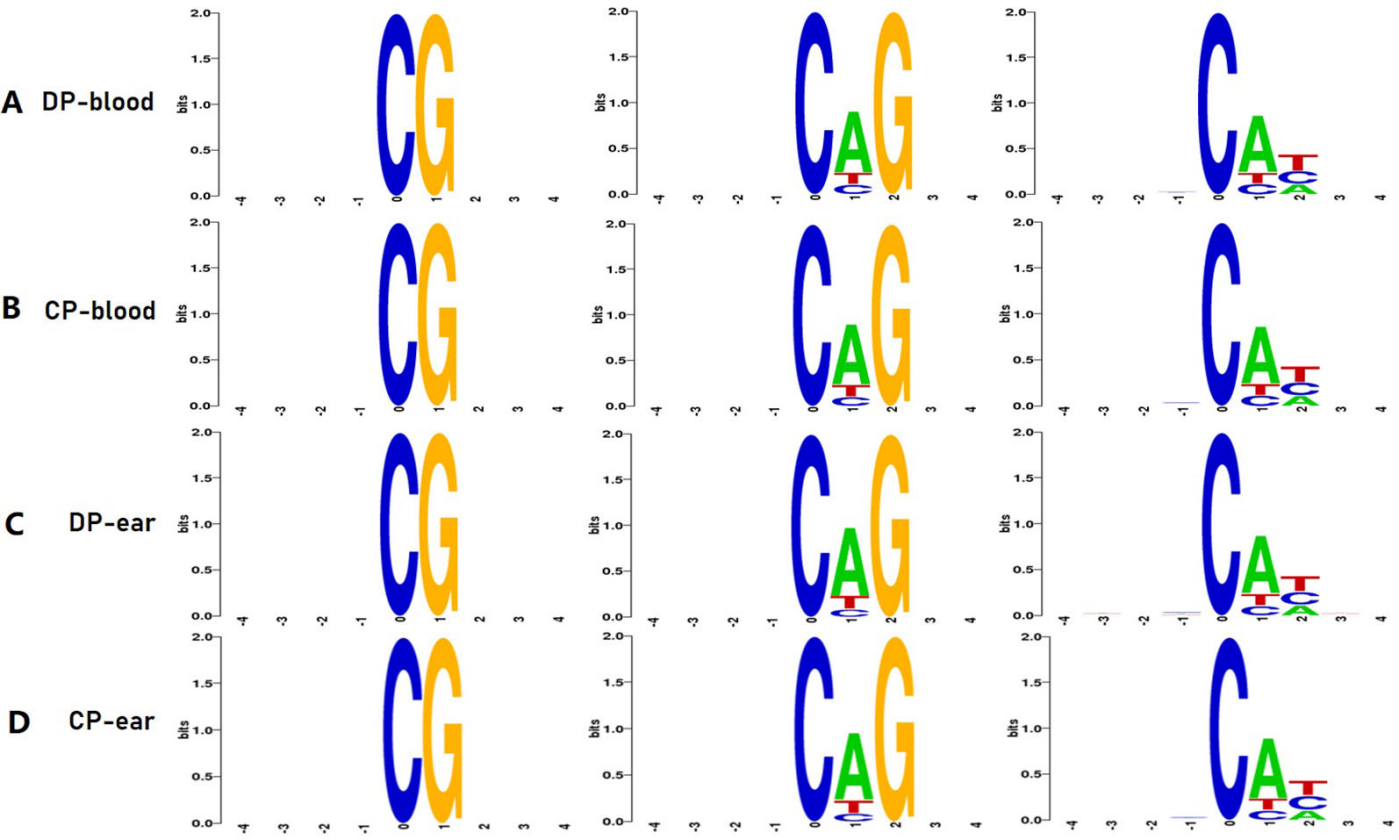
Methylation features of 9 bp spanning 5mC methylcytosine sites. CG, CHG, CHH is the methylation type, H is A, C, or T. Panels **(A–D)** are the 4 bp sequences of the upstream and downstream of the methylated cytosine of DP-blood, CP-blood, DP-ear, and CP-ear, respectively. The abscissa denotes the base number of the methylation site, the total height of each position is the sequence conservation of the base, which represents the relative frequency of the base at that position.

The C bases (CG, CHG, and CHH) of different distribution types had different levels of methylation between different animals and tissues. We counted the distribution of C-base methylation levels for each type using a violin plot. CG methylations were the most abundant, and most of them were hypermethylated with a methylation level greater than 0.7 (**Figure 2A**). The CHG and CHH methylation levels were mostly below 0.2 (**Figures 2B, C**). The chromosomal methylation profile also showed that CG methylation occurred more frequently, and that mCHG and mCHH methylation levels were considerably lower than mCG methylation level, which is consistent with the results of the chromosome methylation map (**Supplementary Figure 1**). Therefore, the methylation status of blood or ear tissue samples and the number of mCs between DP and CP showed existential discrepancy. Next, we analyzed the relationship between sequence context and methylation preference, mapping the 9 bp sequence characteristics around the mCs (**Figure 3**). We observed that the mCG was completely hypermethylated, and that CAG and CAT were the most common motifs of CHG and CHH in the two pigs.

### DNA Methylation of CpG Islands in Functional Regions

**Figure 4:**
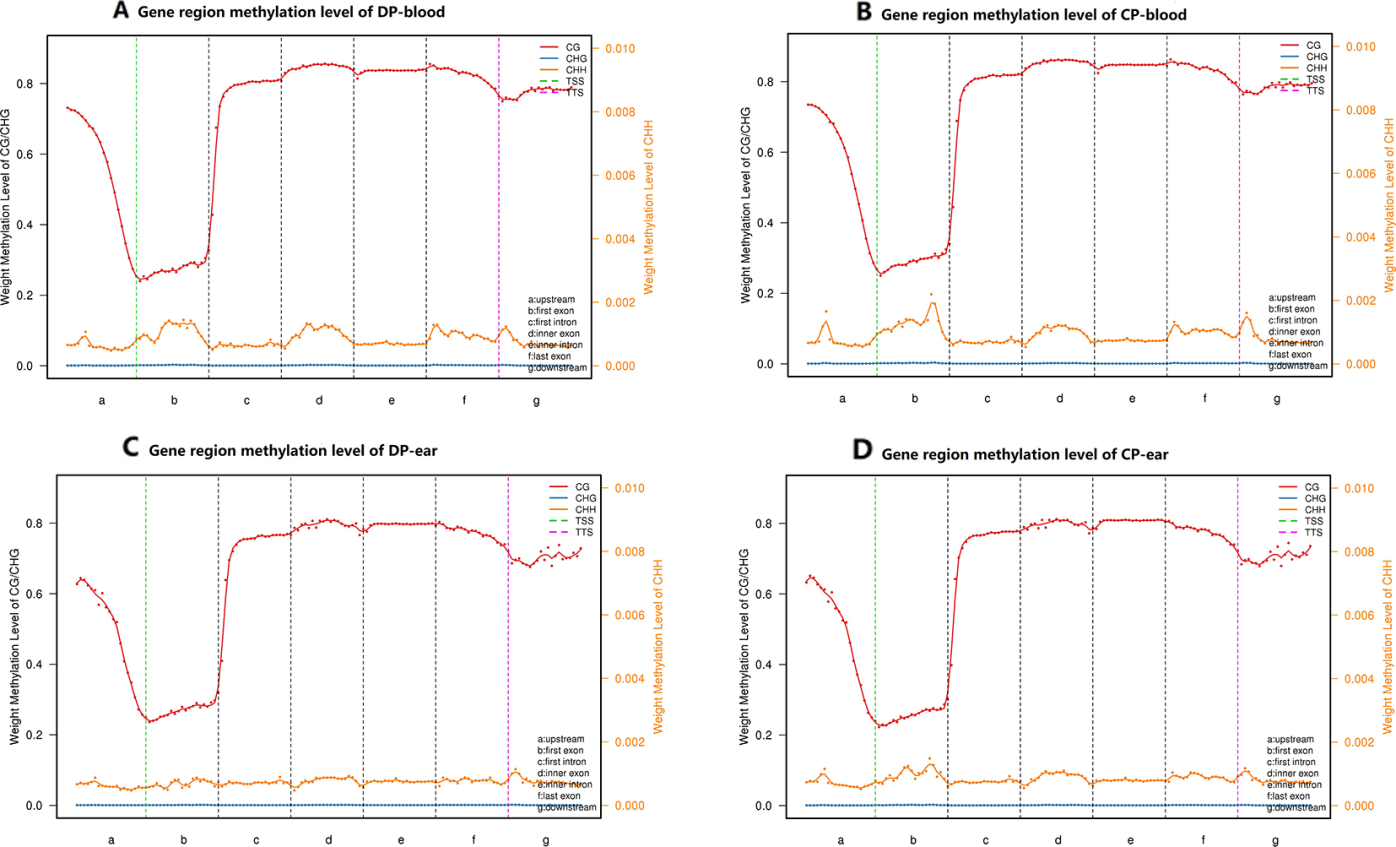
CpG islands distribution in different functional elements. Panels **(A–D)** are CpG islands distribution in the functional elements of DP-blood, CP-blood, DP-ear, and CP-ear, respectively. The methylation region was divided into upstream, general gene body, downstream, TSS and TTS regions. Each region was divided into 20 bins to count weight methylation levels. The abscissa represents the gene functional elements, line color represent methylation type. The left ordinate represents the methylation levels of CG and CHG, and the right ordinate represents the methylation levels of CHH.

We divided all mCs into functional 5′ untranslated region (UTR), first exon, inner exon, first intron, inner intron, last exon, and 3′ UTR. We also measured the methylation of CpG islands in each functional region of the four samples (**Figure 4**). The CpG islands of CG-type methylation in the functional regions of tissues from both pigs were similar. Furthermore, the levels of methylation in the intron regions, exon regions (except the first exon), and downstream were significantly higher than in other regions. CHH-type CpG islands were hypomethylated and stable in each functional region, the level of methylation in the CP was higher than that of the DP, and CHG type CpG islands in the four samples were almost unmethylated.

### Methylation Annotation of the Hypermethylated CpG Islands

**Figure 5:**
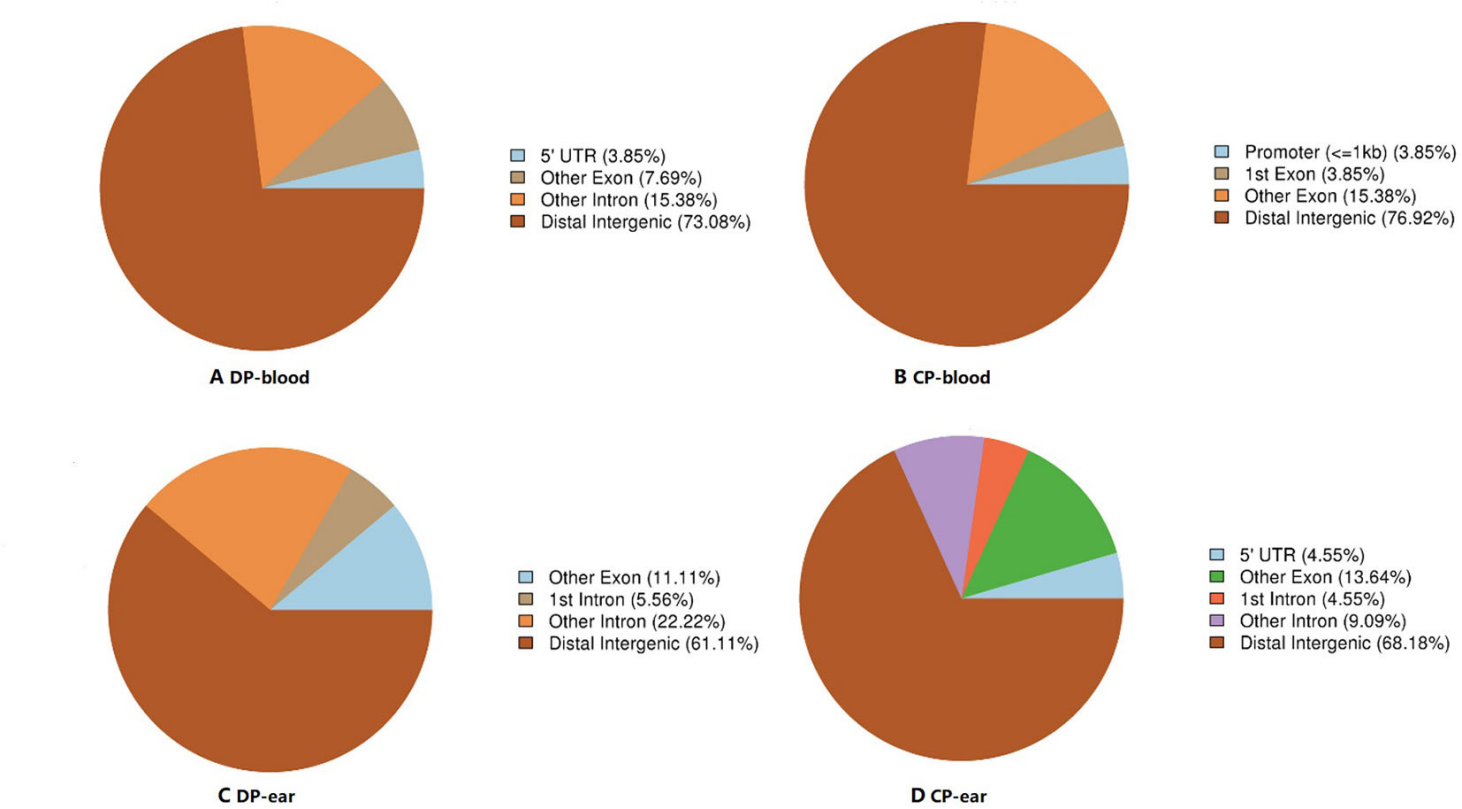
The ratio of hypermethylation CpG islands distribution in different functional elements. Panels **(A, B)** are the CpG islands annotation in functional areas of DP-blood and CP-blood, respectively. Panels **(C, D)** are the CpG islands annotation in functional areas of DP-ear and CP-ear, respectively. The color of the pie chart indicates the functional area and number in parentheses represents the percentage of methylation in the functional area.

We took the 3,000 bp upstream of a gene as promoter region, made an overlap annotation on CpG islands with methylation levels >0.7 and mC coverage >5X, but not including the hypermethylated CpG island with C-degree confidence less than 0.1 (**Figure 5**). In the blood, hypermethylated CpG islands were found in the distal intergenic regions and other exons (except the first exon), but the frequency of methylation were different, while other hypermethylated CpG islands of the two pigs were found in different gene functional regions. In the DP, 73.08% of hypermethylated CpG islands were found at distal intergenic regions, 7.69% of hypermethylated CpG islands were found at other exon (exon region except first exon), 3.85% of hypermethylated CpG islands occurred in the 5′UTR, and 15.38% of hypermethylated CpG islands were found at other introns (intron region except first intron). In contrast, 76.92% of CpG islands were found in distal intergenic regions, 15.38% of hypermethylated CpG islands were found at other exon, 3.85% of hypermethylated CpG islands occurred in a promoter region, and 3.85% of hypermethylated CpG islands were found at first exon regions in the CP. In the ear tissue, hypermethylated CpG islands of the two pigs occurred in the 5′UTR, distal intergenic, intron, and other exon, but their rate of hypermethylation was different. In the DP, 11.11% of hypermethylated CpG islands were found in other exons, 22.22% of hypermethylated CpG islands were found in other introns, 5.56% of hypermethylated CpG islands were found in the first intron, and 61.11% of hypermethylated CpG islands were found in the distal intergenic region. In the CP, these figures were 13.64%, 9.09%, 4.55%, and 68.18%, respectively. Hypermethylated CpG islands were found at distal intergenic region in the DP compared to the CP. There were also some differences in the functional regions and proportions of hypermethylated CpG islands in the two pigs, in which hypermethylated CpG islands in the blood were simultaneously present in the intergenic region and other exons, while the functional regions of hypermethylated CpG islands in the ear tissue were found to be the same just at different proportions.

### Correlation Analysis of DP and CP

**Figure 6:**
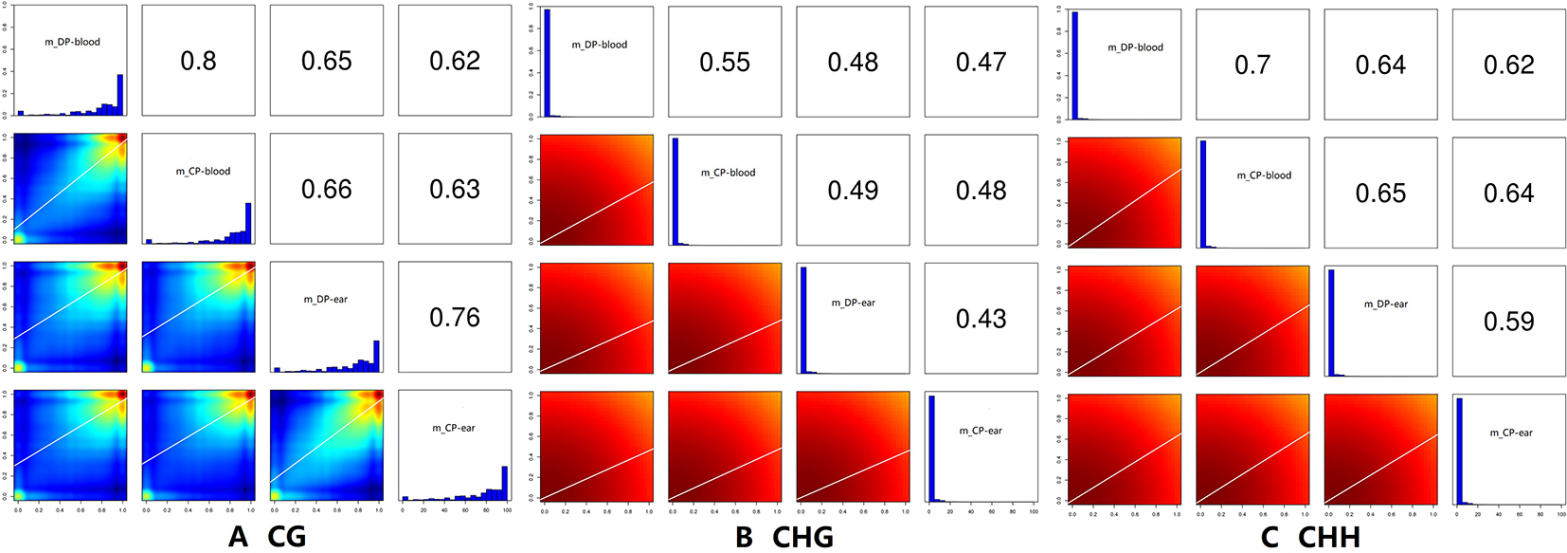
The correlation analysis of samples. The histogram of methylation level in the figure, the abscissa is the methylation level, and the ordinate is proportion of the corresponding methylation level; the value in the figure is the correlation coefficient of two samples. Panels **(A**–**C)** represented the ratio of DMRs in different gene functional regions of CG, CHG, and CHH of the blood, respectively.

Correlation analysis between samples with different types of methylation (**Figure 6**) showed that the correlation coefficient of the CG type of the four samples ranged from 0.63 to 0.80 while CHH type ranged from 0.59 to 0.70, and CHG type ranged from 0.43 to 0.55. Principal component analysis also showed that the type of methylation in each functional region of the four samples was different. Furthermore, CG methylation in the blood samples of the two pigs showed some differences, while CHG methylation between DP-blood sample and CP-ear tissue samples also exhibited some differences. The same was true for CHH methylation of each functional element in DP-blood and CP-ear samples (**Figure 4**).

### Analysis of DMRs

**Figure 7:**
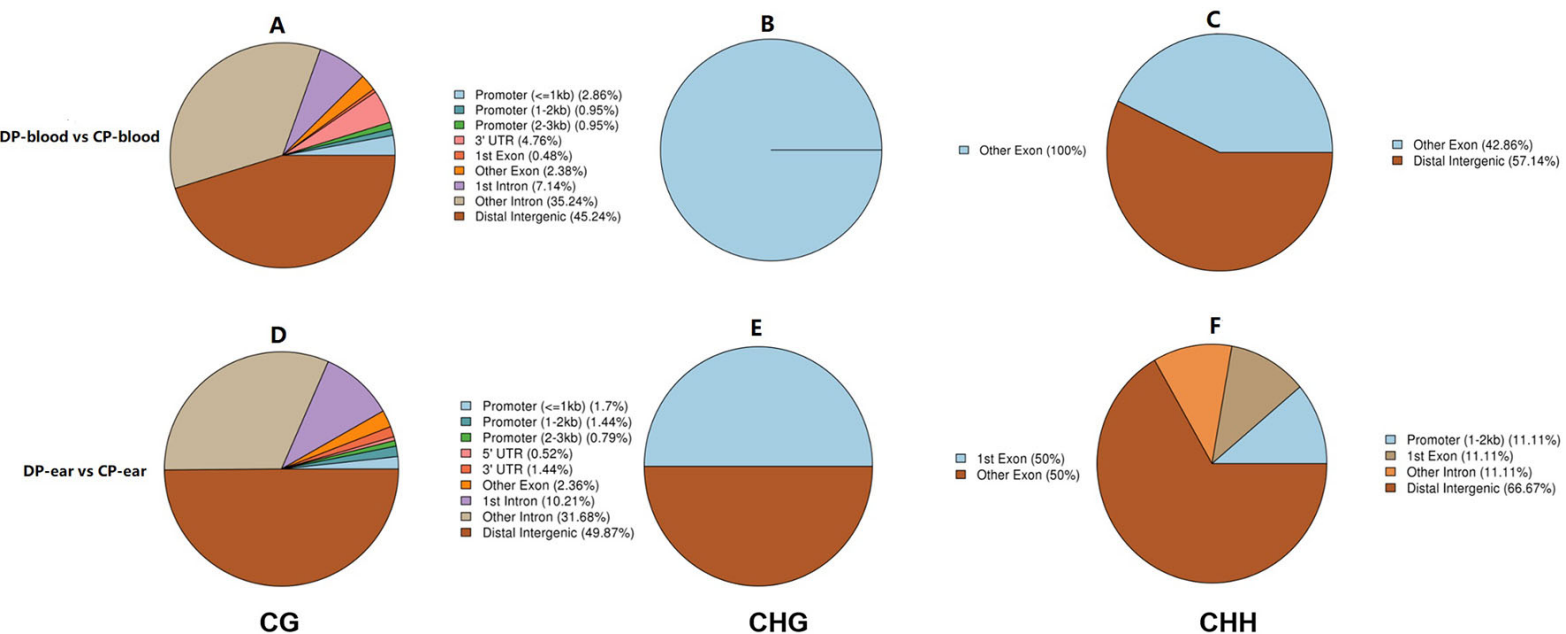
The ratio of DMRs in gene functional regions. Panels **(A–C)** represented the ratio of DMRs in different gene functional regions of CG, CHG, and CHH of the blood, respectively. Panels **(D–F)** represented the ratio of DMRs in different gene functional regions of CG, CHG, and CHH of the ear, respectively.

We annotated the DMGs with gene functional elements (with 3,000 bp 5′ to the TSS and 3′ to transcription termination site as the gene upstream and downstream functional regions, respectively) that associate with DMRs according to the DMR position and genome annotation information (Zhang et al., 2017a). DMRs was annotated according to different methylation types (**Figure 7**). Finally, a total of 210 CG-type, 1 CHG-type, and 7 CHH-type DMRs were identified in all blood samples. Additionally, 764 CG-type, 2 CHG-type, and 9 CHH-type DMRs were identified in the ear tissue samples. Most of these DMRs were located in the gene coding regions and only a few were found in the intergenic regions, in first exon, and in the UTR. The majority of CG type DMRs were found in intergenic regions, second only to intron regions. CHG-type DMRs were mainly located in exons, while CHH-type DMRs were found in the distal intergenic and exon regions. In summary, a total of 215 DMGs were identified in the blood (**Supplementary File 1**) and 707 DMGs in the ear (**Supplementary File 2**).

**Figure 8:**
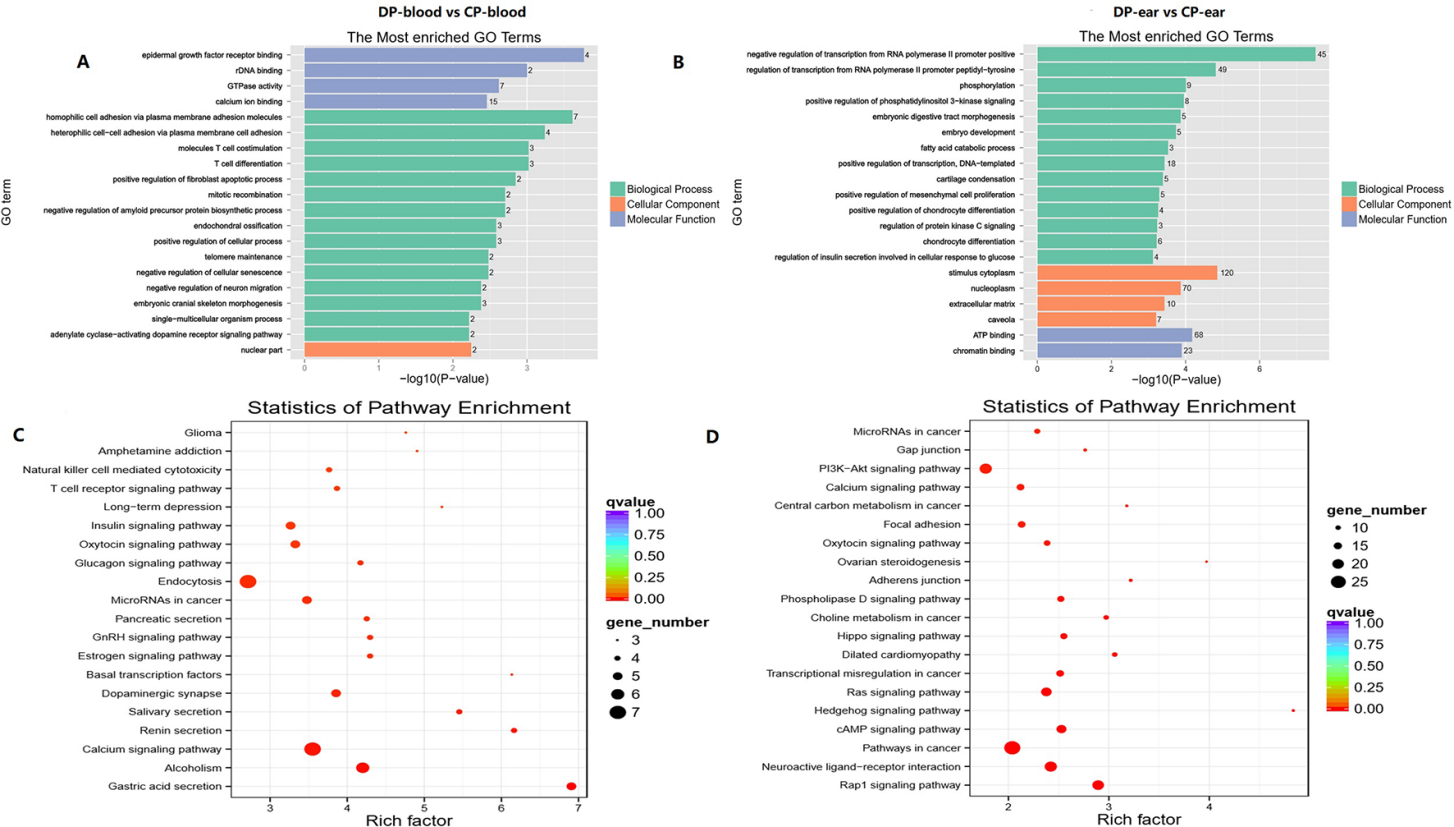
Top GO and top KEGG pathway analysis of CG type DMGs. Panels **(A, C)** were top GO analysis and top KEGG analysis in blood. Panels **(B, D)** were top GO analysis and top KEGG analysis in ear, respectively.

The genes that were located within or closest to the DMRs of the intergenic region were detected and defined as DMGs by ChIPseeker in R. All of the DMGs are enriched in biological processes, cell components, and molecular functions. In the blood, DMGs were enriched in all biological processes except those involved in the rhythmic process, hormone secretion, and cell killing. Cellular components except for the virion, virion part and collagen trimer, molecular functions of antioxidants, receptor regular, and translation regular activity, chemoattractant, and protein tag, in which many DMGs were enriched in immune-related and female reproduction pathways (**Figure 8C, D**). In the ear, DMGs were enriched in all the GO terms except cell killing, molecular function of chemointermediary activity, and translation regular activity. Moreover, many DMGs are enriched on immune-related and reproduction terms (**Figure 8A, B**). KEGG analysis showed that DMGs are mainly enriched on ontogeny, metabolism, and tumor disease, in which many DMGs are enriched in immunity and reproduction associated pathways.

### DMGs Enriched in GO and KEGG Pathways Related to Immunity

To determine whether the cloning process will lead to immune resistance variations between the two pigs, we performed functional enrichment analysis and annotation *via* GO and KEGG for DMGs. Some DMGs are enriched in T/B cell differentiation, thymus development, and other immune-related terms/pathways. In the blood, 16 DMGs are enriched in immunologically related GO terms (**Supplementary File 3**), in which *CARD11*, *CD244*, *RFTN1*, and *THBS1* are enriched in three or more immune-related GO terms. Interestingly, a novel gene (gene ID: *ENSSSCG00000016737*) is enriched in antibiotic response processes. KEGG analysis identified eight DMGs which are enriched in immune-related pathways. *PTPRC*, *GRB2*, and *CARD11* are enriched in three or more immune-related pathways (**Supplementary File 4**), in which a new gene (gene ID: *ENSSSCG00000035088*) associated with B and T cell receptor activity and cytotoxicity of natural killer cells was identified following KEGG analysis. Moreover, *CARD11* and *CD244* are enriched in immune-related terms/pathways. In ear tissue samples, 51 annotated genes and 7 new genes are enriched for the terms “immunity” and “differential methylated” (**Supplementary File 5**). Among these, *IRAK3*, *IL17RA*, *FGR*, *PDE4B*, *CD244*, *TGFB2*, *MX1*, *F2RL1*, *MEF2C*, *GLI3*, *CD83*, *FOXP1*, *CDK6*, *LEP*, *ENSSSCG00000037390*, and *ENSSSCG00000037643* were enriched in three or more immune-related terms. Furthermore, 22 DMGs were enriched in the immunologically related pathways (**Supplementary File 6**) with *ENSSSCG00000039951* and *PTPRC* being enriched in three or more immune-related pathways. *NCK2*, *PLCG1*, *TANK*, *TGFB2*, and *ENSSSCG00000006851* are simultaneously enriched on immune-related terms and pathways.

### DMGs Enriched in GO and KEGG Pathways Related to Reproductive Performance

A total of 55 and 20 DMGs are enriched on oocyte maturation, embryo development, gonad development, estrogen synthesis, and estrogen activity. Additionally, 11 DMGs in blood (**Supplementary File 7**) and 40 DMGs in ear samples (**Supplementary File 8**) are enriched in reproductive related biological processes, whereas seven DMGs in blood (**Supplementary File 9**) and 13 DMGs in ear (**Supplementary File 10**) are enriched in reproductive related pathways. GO analysis has shown that most DMGs are enriched in processed involving embryonic development, in which *NR2F1*, *TUBGCP5*, *RUNX2*, and *ASH2L* are related to steroid hormone signaling, steroid receptor activity, oocyte meiosis cycle, female gonad development, or estrogen responses. In the KEGG analysis, most of the DMGs are mainly enriched in the signaling pathways of oxytocin, estrogen, GnRH, prolactin, and oocyte meiosis; among which, *GANAS* is located in the reproductive-hormone-related pathways and is associated with follicular development. Furthermore, in blood *GNAS* is enriched in post-embryonic body morphogenesis and embryonic hindlimb morphogenesis terms, as well as in ovarian steroidogenesis, oxytocin, estrogen, and GnRH signaling pathways (**Table 3**). *EGFR*, another DMG in ear samples is enriched to embryonic placental development, estrogen, GnRH, and Oxytocin signaling pathways (**Table 3**).

**Table 3:** Ten DMGs associated with immunity or reproduction.

Gene ID	Gene Name	KEGG pathway	GO term
*ENSSSCG00000007520*	*GNAS*	Ovarian steroidogenesis, Oxytocin signaling pathway, Estrogen signaling pathway, GnRH signaling pathway	post-embryonic body morphogenesis, embryonic hindlimb morphogenesis
*ENSSSCG00000022126*	*EGFR*	Estrogen signaling pathway, GnRH signaling pathway, Oxytocin signaling pathway	Embryonic placenta development
*ENSSSCG00000006374*	*CD244*	Natural killer cell mediated cytotoxicity	natural killer cell activation involved in immune response, positive regulation of CD8-positive, α-β T cell proliferation, positive regulation of interferon-γ secretion, positive regulation of interleukin-8 secretion.
*ENSSSCG00000006736*	*CD2*		positive regulation of interferon-γ secretion, positive regulation of interleukin-8 secretion
*ENSSSCG00000021576*	*CD83*		negative regulation of interleukin-4 production, interferon-γ secretion, positive regulation of CD4-positive, alpha-β T cell differentiation, positive regulation of interleukin-10 production, humoral immune response
*ENSSSCG00000013115*	*CD5*		T cell costimulation
*ENSSSCG00000040183*	*CDK6*		response to virus, T cell differentiation in thymus, type B pancreatic cell development, T cell differentiation in thymus, response to virus
*ENSSSCG00000000555*	*ITPR2*	Oocyte meiosis, Oxytocin signaling pathway, Estrogen signaling pathway, GnRH signaling pathway	
*ENSSSCG00000011141*	*CALML5*	Oocyte meiosis, Oxytocin signaling pathway, Estrogen signaling pathway, GnRH signaling pathway	
*ENSSSCG00000021828*	*TAPT1*		embryonic skeletal system development, post-embryonic development, in utero embryonic development

Genes that found within the DMRs or closest to the DMRs of the intergenic region were defined as DMGs to perform KEGG pathway enrichment analysis and gene function enrichment analysis via GO.

## Discussion

### Phenotype and Methylation Were Different Between the CP and DP

Under the same feeding regime and environmental conditions, the first calving age of the CP was 51 days older than the DP, and the litter size of the CP was four piglets less and two live piglets less than the DP in the first two parities and in the first three parities, respectively (**Table 3**). DNA methylation was a common epigenetic modification and affected oocyte meiotic maturation and embryonic development, which in turn affecting the litter size (Gao et al., 2016; Glanzner et al., 2017; Hwang et al., 2017a; Wang et al., 2018). In this study, we found that the level of methylation and the methylated functional regions of the same tissue between the two pigs were different. Furthermore, in both pigs the majority of methylation in the blood occurs in the intergenic region and other exon regions, while other methylations occurred in different regions. In the ear, hypermethylated CpG islands in both animals were found in the same functional region, but the hypermethylation rate of each functional region was different. 3.85% of hypermethylated CpG islands occurred in the promoter regions in the blood of cloned pig, and the proportion of hypermethylated CpG islands occurred in exons was significantly higher than that of donor pigs in blood and ear (with 19.23% vs 7.69% in blood, 13.64% vs 11.11% in ear). This suggested that DNA methylation status in the promoter and exon regions of genes altered in cloned pig, indicating a potential risk of abnormal gene expression thus leading to changes in the cloned pig's phenotype. This was consistent with the results of the DNA methylation of promoter regions were changed in some genes and the DNA methylation status with a trend towards slightly increased methylation levels in cloned pigs (Gao et al., 2011). DNA methylation levels are negatively and monotonically correlated with gene expression levels around the TSS of genes (Zou et al., 2016), this change may lead a poor reproduction performance in the cloned pig. In our study, the DMRs (CP vs DP) suffered more hypomethylation (391/775 DMRs) than hypermethylation (384/775 DMRs) in the ear, suggested the DNA methylation status in the DMRs altered and phenotype changed in cloned pig, this was consistent with the DNA methylation context of skeletal muscle in abnormal and normal cloned pig (Li et al., 2014). Therefore, the methylation context of the ear and blood were different, which was consistent with the results of the DNA methylation in the ovary and intestine of pigs (Yuan et al., 2016). Furthermore, the average sequencing depth was more than 25X, the methylation conversion of reads with a coverage of >10X were more than 87.82% and >74.93% of bases were aligned to the reference genome. In our study, the majority of methylation sites were the CG type and most of them were hypermethylated, this was consistent with the maximum number of CG-type DMRs and DMGs. This shows that we have high quality data allowing us to be confident that our bioinformatic analyses are reliable.

### DMGs Involved in Immunity and Reproduction

This study has identified a number of immune-related regulatory factors such as *CD244*, *CD2*, *CD83*, *CD5*, and *CDK6* with differences in methylation between the CP and the DP. *CD244* is known as an important regulator of various autoimmune defects, where low expression of *CD244* on monocytes can cause systemic lupus erythematosus and the overexpression of *CD244* could accelerate the cancer pathogenesis (Zhang et al., 2017c; Mak et al., 2018). Inhibition of *CD244* expression may promote the expression of IFN-γ/TNF-α in CD8+ T cells and may reduce the incidence and mortality of bovine tuberculosis (Yang et al., 2013; Wang et al., 2015). Binding of *CD244* to *CD2* can reduce the production of antigen-specific interleukin-2 and inhibit the immune response of antiviral *CD8+* T cells (Clarkson and Brown, 2009; Pacheco et al., 2013; McArdel et al., 2016). *CD83*, a specific marker of dendritic cells (DCs) for antigen presentation and lymphocyte activation, can regulate immune responses to prevent colitis by regulating DC surface proteins. *CD83* can also promote the expression of some immune-related genes or the secretion of immune factors (Tze et al., 2011). Found in T lymphocytes, *CD5* is a type of cluster differentiation antigen. High *CD5* expression in peripheral T cells allows them to better respond to foreign antigens and enrich memory populations to produce stronger antigenic responses when exposed to the same antigen again (Freitas et al., 2017). Differences in *CD5* methylation may lead to a change in the expression of *CD5* in the peripheral blood of the CP and DP in this study, which may have had an effect on the immune responses of the two pigs during immunization programs. *CD5* antigen-positive T lymphocytes play a role in signal transduction. These cells work with some immune-related genes and immune factors to maintain immune homeostasis and normal immune function of the thymus (Freitas et al., 2018). Cyclin-dependent kinase, *CDK6*, is a driving factor for cell proliferation and can bind D cell cycle protein 3 to promote tumor cell apoptosis (Wang et al., 2017). A lack of *CDK6* usually leads to anemia, while overexpression leads to hematologic malignancies (Kollmann et al., 2016; Uras et al., 2017). Most of the aforementioned DMGs were enriched in cancer, T/B, NK cell differentiation, and other immune pathways, which are important for the prevention and development of disease (Kaur et al., 2013; Kuwabara et al., 2018; Mazzone et al., 2019). The differences in methylation of these genes may have an effect on immune responses in the CP. However, as the sampled pig or its offspring showed no significant difference in disease characteristics, and the sample size in our study was limited, further investigation is required to confirm this hypothesis.

To explain the difference between the first birth age and total number born (TNB) between the DP and the CP, we annotated the DMGs involved in reproduction. *ITPR2*, *CALML5*, *TAPT1*, *GNAS*, and *EGFR* were enriched in three or more reproduction related pathways (**Table 3**). *ITPR2* is an important regulator of calcium-channel activity, which is the basis of animal fertilization and embryonic development (Kuroda et al., 1999; Miao and Williams, 2012). *TAPT1* defects can lead to fatal chondrodysplasia of the fetus (Symoens et al., 2015) and *CALML5* regulates epidermal differentiation by binding to stratifin (Sun et al., 2015). Differences in the expression of these genes may explain the delayed first calving age and the fewer offspring of the CP. *GNAS* has been shown to have a direct impact on follicular development. Moreover, inhibition of *GNAS* expression improved the success rate of embryonic implantation (Meng et al., 2014). Abnormal methylation of *GNAS* has also been shown to lead to neural tube defects (NTDs) during pregnancy, and even to embryonic development failure (Wang et al., 2017). Differences in *GNAS* expression may explain the differences in the number of litters between the DP and the CP in our study. *EGFR* is involved in the signaling pathways of mature oocytes and embryonic development, and expression of *EGFR* can inhibit oocyte development and maturation by stimulating PI3K/AKT signaling (Park et al., 2017). Inhibition of *EGFR* activates extracellular signal-regulated kinases 1 and 2 (ERK1/2) and accelerates oocyte meiosis (Lei et al., 2018). Conversely, overexpression of *EGFR* promotes progesterone and estrogen secretion thereby obstructing embryonic implantation and development (Wang et al., 2018). Differences in the methylation of *EGFR* may be an important factor in the delayed first calving age and the smaller litter size in the CP. In addition to *ITPR2*, *CALML5*, *TAPT1*, and *GNAS*, many DMGs were enriched in functions related to reproduction or pathways involving oxytocin, prolactin, GnRH, estrogen, ovarian hormones, follicular development, luteinizing hormon (LH) production, gonadal development, or reproductive activities of the females. Consequently, these DMGs may have an impact on the reproductive performance of sows (Napso et al., 2018).

## Conclusion

In this study, we investigated the whole-genome methylation patterns in blood and ear tissue of a CP and a DP. Using this approach, we were able to identify DMGs in both sample types. A total of 60 DMGs were enriched in immune pathways, of which 19 were enriched in more than three immunity pathways. A total of 68 DMGs were enriched in reproductive related pathways, of which 13 participated in more than three pathways related to reproduction. Among which *ITPR2*, *TAPT1*, *GNAS*, and *EGFR* were related to reproduction. Our research provides basic genomic reference for clonal epigenetic research.

## Data Availability Statement

The raw data for this study can be found in the NCBI public database at this URL link: https://www.ncbi.nlm.nih.gov/Traces/study/?acc=PRJNA530044.

## Ethics Statement

This study was carried out in accordance with the recommendations of ' Scientific Ethic Committee of Huazhong Agricultural University (HZAUCA-2016-008), Wuhan, China '. The protocol was approved by the ' Scientific Ethic Committee of Huazhong Agricultural University(HZAUCA-2016-008)&#39.

## Author Contributions

XL, BZ, YM, and SZ designed the experiments. XL, BZ, YM, GM, SF, and MW carried out the experiments. MW, XD, JR, and HW conducted the statistical analysis and discussion. XL, XD, SZ, JR, and MW organized and wrote the manuscript. All the authors have read the paper and have agreed to be co-authors.

## Funding

This work was supported by the Fundamental Research Funds for the Central Universities of China (2662016PY006), National Natural Science Foundation of China (31572375), the Fundamental Research Funds for the Central Universities of China (2662018JC033), the Fundamental Research Funds for the Central Universities (2662017JC027), the National Swine Industry Technology System (No.CARS-35), and Da Bei Nong Group Promoted Project for Young Scholar of HZAU (2017DBN019).

## Conflict of Interest

Authors MW, GM, XL and XD were employed by company Guangxi Yangxiang Co., Ltd, China.

The remaining authors declare that the research was conducted in the absence of any commercial or financial relationships that could be construed as a potential conflict of interest.

## Abbreviations

CP, cloned pig; DMGs, differential methylation genes; DMRs, differential methylation regions; DP, donor pig; SCNT, somatic cell nuclear transplantation.
