# Supplementary material for: Whole-Genome Methylation Analysis Reveals Epigenetic Variation in Cloned and Donor Pigs
Source: Front Genet. 2020 Feb 20;11:23. doi: 10.3389/fgene.2020.00023 (PMC7046149; doi:10.3389/fgene.2020.00023)
Supplement: Supplementary file 1 [file DataSheet_1.zip › Sup Material/Sup File S10.DOCX]

# Supplementary File 10

**DMGs enriched to reproduction related pathways in the ear**

| Gene ID | Gene name | DMG Location | KEGG pathway |
| --- | --- | --- | --- |
| ENSSSCG00000022092 | CYP7B1 | 4: 69,615,559-69,806,496 | Steroid hormone biosynthesis |
| ENSSSCG00000004074 | OPRM1 | 1: 12,634,564-12,702,992 | Estrogen signaling pathway |
| ENSSSCG00000022126 | EGFR | 9: 139,301,586-139,475,037 | Estrogen signaling pathway, GnRH signaling pathway, Oxytocin signaling pathway |
| ENSSSCG00000027952 | ADCY5 | 13:136,973,000-137,133,977 | Estrogen signaling pathway, Oocyte meiosis, GnRH signaling pathway, Oxytocin signaling pathway, Progesterone-mediated oocyte maturation, Ovarian steroidogenesis |
| ENSSSCG00000031717 | ADCY8 | 4: 9,645,797-9,871,259 | Estrogen signaling pathway, Oocyte meiosis, GnRH signaling pathway, Oxytocin signaling pathway, Progesterone-mediated oocyte maturation, Ovarian steroidogenesis |
| ENSSSCG00000032214 | novel gene | 1: 152,593,651-152,636,635 | Prolactin signaling pathway |
| ENSSSCG00000033473 | novel gene | 5: 99,451,960-99,452,596 | Oxytocin signaling pathway |
| ENSSSCG00000033962 | novel gene | 1: 20,890,274-20,890,516 | Estrogen signaling pathway, Oocyte meiosis, GnRH signaling pathway, Oxytocin signaling pathway |
| ENSSSCG00000034072 | novel gene | 5: 80,521,815-80,531,021 | Estrogen signaling pathway |
| ENSSSCG00000036255 | CACNG3 | 3: 21,853,311-21,960,144 | Oxytocin signaling pathway |
| ENSSSCG00000036874 | MYLK3 | 6: 37,850,754-37,920,754 | Oxytocin signaling pathway |
| ENSSSCG00000038694 | CCND2 | 5: 66,087,379-66,114,616 | Prolactin signaling pathway |
| ENSSSCG00000039947 | KCNJ2 | 12: 10,351,715-10,362,244 | Oxytocin signaling pathway |

Genes that located within the differential methylation regions or closest to the differential methylation regions of the intergenic region were defined as DMGs to perform KEGG pathway enrichment analysis.
