# Supplementary material for: Whole-Genome Methylation Analysis Reveals Epigenetic Variation in Cloned and Donor Pigs
Source: Front Genet. 2020 Feb 20;11:23. doi: 10.3389/fgene.2020.00023 (PMC7046149; doi:10.3389/fgene.2020.00023)
Supplement: Supplementary file 1 [file DataSheet_1.zip › Sup Material/Sup File S3.DOCX]

# Supplementary File 3

**DMGs enriched to immunity related terms in the blood**

| Gene ID | Gene name | DMG Location | | GO Names |
| --- | --- | --- | --- | --- |
| *ENSSSCG00000011810* | *BCL6* | 13:125,377,250-125,428,606 | B cell differentiation | |
| *ENSSSCG00000034390* | *CARD11* | 3:2145180-2266355:-1 | | Regulation of immune response，positive regulation of T cell proliferation，positive regulation of interleukin-2 biosynthetic process，regulation of T cell differentiation，T cell costimulation |
| *ENSSSCG00000006374* | *CD244* | 4:89668249-89700971:1 | | Natural killer cell activation involved in immune response，positive regulation of interferon-gamma secretion，positive regulation of interleukin-8 secretion |
| *ENSSSCG00000013115* | *CD5* | 2:10644686-10673958:-1 | | T cell costimulation |
| *ENSSSCG00000040183* | *CDK6* | 9:72518100-72765558:-1 | | Response to virus，T cell differentiation in thymus |
| *ENSSSCG00000006231* | *CHD7* | 4:72573566-72694141:-1 | | T cell differentiation |
| *ENSSSCG00000015476* | *CHI3L1* | 9:113882214-113895510:-1 | | Response to interleukin-6，interleukin-8 secretion |
| *ENSSSCG00000015235* | *ETS1* | 9:55377266-55512247:-1 | | response to antibioticresponse |
| *ENSSSCG00000017206* | *GRB2* | 12:5940645-6010836:1 | | T cell costimulation |
| *ENSSSCG00000008573* | *RAB10* | 3:112841701-112924314:-1 | | Antigen processing and presentation |
| *ENSSSCG00000011198* | *RFTN1* | 13:3454449-3675078:-1 | | Interleukin-17 production，toll-like receptor 3 signaling pathway，T cell antigen processing and presentation |
| *ENSSSCG00000001710* | *RUNX2* | 7:40106513-40349398:1 | | T cell differentiation |
| *ENSSSCG00000038062* | *SAMSN1* | 13:179427521-179478876:-1 | | Negative regulation of B cell activation，negative regulation of adaptive immune response |
| *ENSSSCG00000027855* | *SOCS1* | 3:31881891-31882722:1 | | regulation of Interferon-gamma-mediated signaling pathway |
| *ENSSSCG00000001081* | *SOX4* | 7:16891319-16892752:1 | | T cell differentiation |
| *ENSSSCG00000004789* | *THBS1* | 1:131728784-131746115:-1 | | Immune response，negative regulation of interleukin-12 production，negative Regulation of antigen processing and presentation of peptide or polysaccharide antigen via MHC class II |
| *ENSSSCG00000016737* | *Novel gene* | 18:50561926-50566861:-1 | | Response to antibiotic |

Genes that located within the differential methylation regions or closest to the differential methylation regions of the intergenic region were defined as DMGs to perform gene function enrichment analysis via Gene Ontology (GO).
