# Supplementary material for: Whole-Genome Methylation Analysis Reveals Epigenetic Variation in Cloned and Donor Pigs
Source: Front Genet. 2020 Feb 20;11:23. doi: 10.3389/fgene.2020.00023 (PMC7046149; doi:10.3389/fgene.2020.00023)
Supplement: Supplementary file 1 [file DataSheet_1.zip › Sup Material/Sup File S4.DOCX]

# Supplementary File 4

**DMGs enriched to immunity related pathways in the blood.**

| Gene ID | Gene name | DMG Location | KEGG pathway |
| --- | --- | --- | --- |
| *ENSSSCG00000006374* | *CD244* | 4:89668249-89700971:1 | Natural killer cell mediated cytotoxicity |
| *ENSSSCG00000010908* | *PTPRC* | 10:21483275-21602035:1 | Primary immunodeficiency, Fc gamma R-mediated phagocytosis, T cell receptor signaling pathway |
| *ENSSSCG00000017206* | *GRB2* | 12:5940645-6010836:1 | Acute myeloid leukemia, B/T cell receptor signaling pathway, Natural killer cell mediated cytotoxicity, Jak-STAT signaling pathway |
| *ENSSSCG00000023301* | *F13B* | AEMK02000698.1:2581987-2612598:1 | Complement and coagulation cascades |
| *ENSSSCG00000027855* | *SOCS1* | 3:31881891-31882722:1 | Jak-STAT signaling pathway |
| *ENSSSCG00000034390* | *CARD11* | 3:2145180-2266355:-1 | NF-kappa B signaling pathway, B,T cell receptor signaling pathway |
| *ENSSSCG00000035088* | *Novel gene* | 8:119587111-119739310:-1 | B,T cell receptor signaling pathway, Natural killer cell mediated cytotoxicity |
| *ENSSSCG00000007797* | *ITGAL* | 3:17817568-17858086:-1 | Natural killer cell mediated cytotoxicity |

Genes that located within the differential methylation regions or closest to the differential methylation regions of the intergenic region were defined as DMGs to perform KEGG pathway enrichment analysis.
