# Supplementary material for: Whole-Genome Methylation Analysis Reveals Epigenetic Variation in Cloned and Donor Pigs
Source: Front Genet. 2020 Feb 20;11:23. doi: 10.3389/fgene.2020.00023 (PMC7046149; doi:10.3389/fgene.2020.00023)
Supplement: Supplementary file 1 [file DataSheet_1.zip › Sup Material/Sup File S5.DOCX]

Supplementary File 5: DMGs enriched to immunity related terms in the ear.

| Gene ID | Gene name | DMG Location | GO Names |
| --- | --- | --- | --- |
| *ENSSSCG00000000475* | *IRAK3* | 5:30555108-30598954:1 | negative regulation of innate immune response, positive regulation of NF-kappaB transcription factor activity, negative regulation of toll-like receptor signaling pathway, negative regulation of macrophage cytokine production, interleukin-1-mediated signaling pathway, negative regulation of interleukin-12/6 production, negative regulation of NF-kappaB transcription factor activity, negative regulation of interleukin-6 production |
| *ENSSSCG00000000765* | *IL17RA* | 5:69461529-69480112:1 | positive regulation of interleukin-13，5 secretion，positive regulation of interleukin-23 production，defense response to fungus |
| *ENSSSCG00000000875* | *NR1H4* | 5:83607225-83683086:-1 | innate immune response |
| *ENSSSCG00000001027* | *BMP6* | 7:5058036-5211316:1 | immune response，type B pancreatic cell development |
| *ENSSSCG00000001033* | *SLC35B3* | 7:5581756-5606551:-1 | positive ，negative regulation of defense response to virus by host |
| *ENSSSCG00000001061* | *JARID2* | 7:11303859-11602097:1 | thymus development |
| *ENSSSCG00000001554* | *SRPK1* | 7:31645755-31701405:-1 | positive regulation of viral genome replication |
| *ENSSSCG00000002965* | *ACTN4* | 6:47446034-47569609:1 | positive regulation of NIK/NF-kappaB signaling |
| *ENSSSCG00000003578* | *FGR* | 6:84809531-84830399:-1 | defense response to Gram-positive bacterium，regulation of phagocytosis，regulation of innate immune response |
| *ENSSSCG00000003805* | *PDE4B* | 6:146166328-146373149:-1 | interferon-gamma secretion and positive regulatio，T cell receptor signaling pathway |
| *ENSSSCG00000003973* | *CTPS1* | 6:170201751-170233074:-1 | B，T cell proliferation |
| *ENSSSCG00000004369* | *PRDM1* | 1:72312082-72337411:1 | negative regulation of B cell proliferation |
| *ENSSSCG00000005240* | *DOCK8* | 1:221256607-221492199:-1 | memory T cell proliferation，negative regulation of T cell apoptotic process |
| *ENSSSCG00000006374* | *CD244* | 4:89668249-89700971:1 | natural killer cell activation involved in immune response，positive regulation of CD8-positive, alpha-beta T cell proliferation，positive regulation of interferon-gamma secretion，positive regulation of interleukin-8 secretion |
| *ENSSSCG00000006588* | *S100A9* | 4:96235327-96239285:-1 | innate immune response，leukocyte migration involved in inflammatory response |
| *ENSSSCG00000006736* | *CD2* | 4:103962527-103977913:-1 | positive regulation of interferon-gamma secretion，positive regulation of interleukin-8 secretion |
| *ENSSSCG00000006851* | *Novel gene* | 4:112006770-112023016:1 | positive regulation of B cell proliferation |
| *ENSSSCG00000007356* | *PLCG1* | 17:43810032-43844551:1 | T cell receptor signaling pathway |
| *ENSSSCG00000008144* | *NCK2* | 3:49063816-49199320:-1 | positive regulation of T cell proliferation |
| *ENSSSCG00000008619* | *DDX1* | 3:121821792-121856601:-1 | response to virus |
| *ENSSSCG00000008645* | *ID2* | 3:127499410-127503297:-1 | negative regulation of B cell differentiation |
| *ENSSSCG00000009480* | *NDFIP2* | 11:51493976-51560303:1 | positive regulation of I-kappaB kinase/NF-kappaB signaling |
| *ENSSSCG00000010816* | *TGFB2* | 10:8305539-8405771:1 | negative regulation of macrophage cytokine production，positive regulation of immune response，positive regulation of activation-induced cell death of T cells |
| *ENSSSCG00000011111* | *CAMK1D* | 10:59205631-59630946:-1 | positive regulation of phagocytosis |
| *ENSSSCG00000011201* | *SATB1* | 13:5317829-5392984:-1 | CD4-positive, alpha-beta T cell differentiation，activated T cell proliferation |
| *ENSSSCG00000011538* | *LMCD1* | 13:64933651-64996454:1 | positive regulation of defense response to virus by host |
| *ENSSSCG00000012077* | *MX1* | 13:204847561-204868477:1 | response to type III interferon，negative regulation of viral genome replication，cellular response to type I interferon，defense response to virus |
| *ENSSSCG00000012594* | *IL13RA2* | X:94401713-94453171:-1 | immunoglobulin mediated immune response |
| *ENSSSCG00000013302* | *CAT* | 2:26493626-26581452:-1 | Positive，negative regulation of NF-kappaB transcription factor activity |
| *ENSSSCG00000014091* | *F2RL1* | 2:85732082-85747621:1 | interferon-gamma secretion, positive regulation of phagocytosis, engulfment, positive regulation of interleukin-6 secretion, positive regulation of toll-like receptor 2 signaling pathway, positive regulation of I-kappaB kinase/NF-kappaB signaling，T cell activation involved in immune response，positive regulation of cytokine secretion involved in immune response，negative/positive regulation of toll-like receptor 3 signaling pathway, interleukin-1B/10 secretion, defense response to virus, leukocyte proliferation, positive regulation of interleukin-8 secretion, positive regulation of toll-like receptor 4 signaling pathway |
| *ENSSSCG00000014149* | *MEF2C* | 2:96122044-96296902:-1 | positive regulation of B cell proliferation, humoral immune response, response to virus |
| *ENSSSCG00000014364* | *ANKHD1* | 2:142166314-142298097:1 | innate immune response |
| *ENSSSCG00000015889* | *TANK* | 15:67994692-68048011:1 | I-kappaB kinase/NF-kappaB signaling |
| *ENSSSCG00000016763* | *GLI3* | 18:52403160-52695397:1 | negative regulation of alpha-beta T cell differentiation, thymocyte apoptotic process, negative thymic T cell selection, positive regulation of alpha-beta T cell differentiation |
| *ENSSSCG00000017251* | *SOX9* | 12:8641629-8647764:-1 | negative regulation of immune system process |
| *ENSSSCG00000021576* | *CD83* | 7:10359211-10387002:1 | negative regulation of interleukin-4 production, interferon-gamma secretion, positive regulation of CD4-positive, alpha-beta T cell differentiation, positive regulation of interleukin-10 production, humoral immune response |
| *ENSSSCG00000022448* | *CLNK* | 8:6609051-6770094:-1 | immune response |
| *ENSSSCG00000023709* | *PTPRJ* | 2:14549537-14726715:-1 | negative regulation of T cell receptor signaling pathway |
| *ENSSSCG00000024161* | *Novel gene* | 7:22972173-22976930:1 | immune response |
| *ENSSSCG00000024311* | *CCR2* | 13:29368747-29376350:1 | immune response |
| *ENSSSCG00000024392* | *THEMIS* | 1:35333473-35521353:1 | T cell receptor signaling pathway, positive T cell selection |
| *ENSSSCG00000024777* | *HDAC4* | 15:138381635-138657270:-1 | response to interleukin-1 |
| *ENSSSCG00000027251* | *CDC7* | 4:125429530-125459489:-1 | phagocytosis |
| *ENSSSCG00000027675* | *FOXP1* | 13:52348094-52974267:-1 | interleukin-21 secretion, positive regulation of immunoglobulin production, immunoglobulin V(D)J recombination, pre-B cell differentiation |
| *ENSSSCG00000028157* | *CASP8* | 15:104923429-104951747:1 | Negative/positive regulation of I-kappaB kinase/NF-kappaB signaling, |
| *ENSSSCG00000028304* | *ZFP36L1* | 7:92488010-92495427:-1 | T cell differentiation in thymus |
| *ENSSSCG00000028806* | *RPS6KA4* | 2:7751967-7765306:-1 | interleukin-1-mediated signaling pathway |
| *ENSSSCG00000031846* | *Novel gene* | 2:144907626-144911544:1 | positive regulation of I-kappaB kinase/NF-kappaB signaling, positive regulation of NF-kappaB transcription factor activity |
| *ENSSSCG00000032214* | *Novel gene* | 1:152593651-152636635:-1 | negative regulation of T cell activation |
| *ENSSSCG00000032320* | *TCIM* | 17:9441482-9443050:1 | endothelial cell activation involved in immune response |
| *ENSSSCG00000033175* | *SASH1* | 1:17306373-17662825:-1 | positive regulation of NIK/NF-kappaB signaling |
| *ENSSSCG00000034610* | *Novel gene* | 5:30188571-30332703:1 | negative regulation by host of viral transcription |
| *ENSSSCG00000035972* | *TULP1* | 7:31362874-31379114:-1 | positive regulation of phagocytosis, phagocytosis, recognition |
| *ENSSSCG00000037390* | *Novel gene* | 1:228956158-228956415:-1 | T cell proliferation involved in immune response, T cell differentiation in thymus, activation-induced cell death of T cells |
| *ENSSSCG00000037643* | *Novel gene* | 6:146614846-146616663:-1 | positive regulation of interleukin-2 production, T cell receptor signaling pathway, positive regulation of interferon-gamma production |
| *ENSSSCG00000040183* | *CDK6* | 9:72518100-72765558:-1 | type B pancreatic cell development, T cell differentiation in thymus, response to virus |
| *ENSSSCG00000040333* | *NUP62* | 6:54921627-54923227:-1 | positive regulation of I-kappaB kinase/NF-kappaB signaling |
| *ENSSSCG00000040464* | *LEP* | 18:20106179-20123451:-1 | interleukin-6,8 secretion, phagocytosis, positive regulation of T cell proliferation, interleukin-12 production |

Genes that located within the differential methylation regions or closest to the differential methylation regions of the intergenic region were defined as DMGs to perform gene function enrichment analysis via Gene Ontology (GO).
