# Supplementary material for: Whole-Genome Methylation Analysis Reveals Epigenetic Variation in Cloned and Donor Pigs
Source: Front Genet. 2020 Feb 20;11:23. doi: 10.3389/fgene.2020.00023 (PMC7046149; doi:10.3389/fgene.2020.00023)
Supplement: Supplementary file 1 [file DataSheet_1.zip › Sup Material/Sup File S6.DOCX]

# Supplementary File 6

**DMGs enriched to immunity related pathways in the ear**

| Gene ID | Gene Name | DMG Location | KEGG Pathway |
| --- | --- | --- | --- |
| *ENSSSCG00000006851* | *Novel gene* | 4:112006770-112023016:1 | B cell receptor signaling pathway |
| *ENSSSCG00000039951* | *Novel gene* | 5:94229805-94232495:1 | B cell receptor signaling pathway, Primary immunodeficiency, Intestinal immune network for IgA production, Fc gamma R-mediated phagocytosis, Autoimmune thyroid disease, Primary immunodeficiency |
| *ENSSSCG00000024310* | *F13A1* | 7:3750642-3901444:-1 | Complement and coagulation cascades |
| *ENSSSCG00000025463* | *PROS1* | 13:165914997-165995755:1 | Complement and coagulation cascades, |
| *ENSSSCG00000010908* | *PTPRC* | 10:21483275-21602035:1 | Primary immunodeficiency, Fc gamma R-mediated phagocytosis, T cell receptor signaling pathway |
| *ENSSSCG00000003839* | *Novel gene* | 6:155800840-155894915:1 | Fc gamma R-mediated phagocytosis, |
| *ENSSSCG00000007356* | *PLCG1* | 17:43810032-43844551:1 | Fc gamma R-mediated phagocytosis, T cell receptor signaling pathway, |
| *ENSSSCG00000009300* | *WASF3* | 11:4322893-4363618:1 | Fc gamma R-mediated phagocytosis, |
| *ENSSSCG00000028759* | *ASAP1* | 4:10103193-10451924:1 | Fc gamma R-mediated phagocytosis |
| *ENSSSCG00000008841* | *FDGFRA* | 8:40977604-41018506:1 | HTLV-I infection |
| *ENSSSCG00000009751* | *FZD10* | 14:24920679-24922424:-1 | HTLV-I infection |
| *ENSSSCG00000010816* | *TGFB2* | 10:8305539-8405771:1 | HTLV-I infection |

Genes that located within the differential methylation regions or closest to the differential methylation regions of the intergenic region were defined as DMGs to perform KEGG pathway enrichment analysis.
