# Supplementary material for: Whole-Genome Methylation Analysis Reveals Epigenetic Variation in Cloned and Donor Pigs
Source: Front Genet. 2020 Feb 20;11:23. doi: 10.3389/fgene.2020.00023 (PMC7046149; doi:10.3389/fgene.2020.00023)
Supplement: Supplementary file 1 [file DataSheet_1.zip › Sup Material/Sup File S7.DOCX]

# Supplementary File 7

DMGs enriched to reproduction related terms in the blood.

| Gene ID | Gene name | DMG Location | GO Names |
| --- | --- | --- | --- |
| *ENSSSCG00000014157* | *NR2F1* | 2: 100,447,904-100,457,904 | steroid hormone mediated signaling pathway, steroid hormone receptor activity |
| *ENSSSCG00000027105* | *TUBGCP5* | 15: 31,995,072-32,041,983 | meiotic cell cycle |
| *ENSSSCG00000017693* | *AATF* | 12: 38,461,807-38,563,539 | embryonic cleavage |
| *ENSSSCG00000032315* | *XRCC2* | 18: 4,808,467-4,835,802 | in utero embryonic development |
| *ENSSSCG00000006231* | *CHD7* | 4: 72,573,566-72,694,141 | embryonic hindlimb morphogenesis, in utero embryonic development |
| *ENSSSCG00000007520* | *GNAS* | 17: 58,998,981-59,055,340 | post-embryonic body morphogenesis, embryonic hindlimb morphogenesis |
| *ENSSSCG00000016160* | *ERBB4* | 15:113,974,697-114,696,336 | embryonic pattern specification |
| *ENSSSCG00000003778* | *LHX8* | 6: 138,075,385-138,102,301 | embryonic forelimb morphogenesis |
| *ENSSSCG00000001710* | *RUNX2* | 7: 40,106,513-40,349,398 | female genitalia development |
| *ENSSSCG00000021898* | *ASH2L* | 15: 48,385,960-48,417,543 | response to estrogen |
| *ENSSSCG00000006760* | *HIPK1* | 4: 106,566,986-106,627,413 | embryonic retina morphogenesis in camera-type eye |

Genes that located within the differential methylation regions or closest to the differential methylation regions of the intergenic region were defined as DMGs to perform gene function enrichment analysis via Gene Ontology (GO).
