# Supplementary material for: Whole-Genome Methylation Analysis Reveals Epigenetic Variation in Cloned and Donor Pigs
Source: Front Genet. 2020 Feb 20;11:23. doi: 10.3389/fgene.2020.00023 (PMC7046149; doi:10.3389/fgene.2020.00023)
Supplement: Supplementary file 1 [file DataSheet_1.zip › Sup Material/Sup File S8.DOCX]

# Supplementary File 8

**DMGs enriched to reproduction related terms in the ear**

| Gene ID | Gene name | DMG Location | GO **Names** |
| --- | --- | --- | --- |
| *ENSSSCG00000008606* | *OSR1* | 3: 118,597,516-118,765,104 | embryonic forelimb morphogenesis, embryonic digit morphogenesis, embryonic skeletal limb joint morphogenesis, embryonic hindlimb morphogenesis, gonad development |
| *ENSSSCG00000027312* | *PTCH1* | 10: 26,626,659-26,703,223 | embryonic organ development, in utero embryonic development, embryonic limb morphogenesis |
| *ENSSSCG00000021828* | *TAPT1* | 8:11,365,423..11,415,699 | embryonic skeletal system development, post-embryonic development, in utero embryonic development |
| *ENSSSCG00000015780* | *STOX2* | 15: 45,084,682-45,299,061 | embryo development |
| *ENSSSCG00000024373* | *TRIP12* | 15:130,787,843-130,939,825 | embryo development |
| *ENSSSCG00000017730* | *CDK5R1* | 12: 42,493,321-42,494,659 | embryo development |
| *ENSSSCG00000024960* | *PDGFC* | 8: 45,578,002-45,808,789 | embryo development |
| *ENSSSCG00000035721* | *Novel gene* | 11: 1,539,207-1,592,813 | embryo development |
| *ENSSSCG00000013566* | *INSR* | 2: 71,797,125-71,936,215 | regulation of female gonad development，regulation of embryonic development |
| *ENSSSCG00000036611* | *NPVF* | 18: 47,072,644-47,087,357 | negative regulation of gonadotropin secretion |
| *ENSSSCG00000014149* | *MEF2C* | 2: 96,122,044-96,296,902 | embryonic viscerocranium morphogenesis |
| *ENSSSCG00000025592* | *TP63* | 13: 127,116,105-127,347,070 | female genitalia morphogenesis, embryonic limb morphogenesis |
| *ENSSSCG00000000875* | *NR1H4* | 5: 83,607,225-83,683,086 | steroid hormone mediated signaling pathway |
| *ENSSSCG00000010698* | *FGFR2* | 14:131,181,713-131,289,414 | post-embryonic development, embryonic digestive tract morphogenesis, embryonic pattern specification |
| *ENSSSCG00000004587* | *MYO1E* | 1: 112,677,820-112,901,999 | post-embryonic development, in utero embryonic development |
| *ENSSSCG00000016763* | *GLI3* | 18: 52,403,160-52,695,397 | embryonic digit morphogenesis, in utero embryonic development, embryonic skeletal system morphogenesis, |
|  |  |  | embryonic digestive tract morphogenesis |
| *ENSSSCG00000008841* | *PDGFRA* | 8: 40,977,604-41,018,506 | in utero embryonic development, embryonic digestive tract morphogenesis |
| *ENSSSCG00000003774* | *Novel gene* | 6: 137,410,919-137,453,959 | ovarian follicle development, female gamete generation |
| *ENSSSCG00000008842* | *KIT* | 8: 41,402,322-41,492,977 | ovarian follicle development, embryonic hemopoiesis |
| *ENSSSCG00000034191* | *SOX6* | 2: 42,452,778-43,068,323 | post-embryonic development, in utero embryonic development |
| *ENSSSCG00000004369* | *PRDM1* | 1: 72,312,082-72,337,411 | post-embryonic development, germ cell development |
| *ENSSSCG00000029621* | *BMPR1B* | 8: 124,535,864-124,841,274 | ovarian cumulus expansion |
| *ENSSSCG00000016639* | *FOXP2* | 18: 31,339,000-31,458,143 | post-embryonic development |
| *ENSSSCG00000016291* | *GIGYF2* | 15: 133,180,522-133,311,516 | post-embryonic development |
| *ENSSSCG00000010816* | *TGFB2* | 10: 8,305,539-8,405,771 | embryonic digestive tract development |
| *ENSSSCG00000000103* | *DMC1* | 5: 9,433,058-9,470,549 | oocyte maturation，ovarian follicle development |
| *ENSSSCG00000017298* | *TANC2* | 12: 15,454,242-15,813,847 | in utero embryonic development |
| *ENSSSCG00000007356* | *PLCG1* | 17: 43,810,032-43,844,551 | in utero embryonic development |
| *ENSSSCG00000009031* | *EDNRA* | 8: 81,210,225-81,277,066 | in utero embryonic development |
| *ENSSSCG00000010329* | *ZMIZ1* | 14: 81,536,419-81,644,262 | in utero embryonic development |
| *ENSSSCG00000027675* | *FOXP1* | 13: 52,348,094-52,974,267 | in utero embryonic development |
| *ENSSSCG00000000103* | *DMC1* | 5: 9,433,058-9,470,549 | ovarian follicle development |
| *ENSSSCG00000008645* | *ID2* | 3: 127,499,410-127,503,297 | embryonic digestive tract morphogenesis |
| *ENSSSCG00000008649* | *SOX11* | 3: 129,798,854-129,800,197 | embryonic digestive tract morphogenesis |
| *ENSSSCG00000022126* | *EGFR* | 9: 139,301,586-139,475,037 | embryonic placenta development |
| *ENSSSCG00000008813* | *CORIN* | 8: 37,530,213-37,809,567 | female pregnancy |
| *ENSSSCG00000033314* | *DLX6* | 9: 76,623,661-76,633,291 | embryonic limb morphogenesis |
| *ENSSSCG00000029803* | *KDM2B* | 14: 31,057,005-31,203,505 | embryonic camera-type eye morphogenesis |
| *ENSSSCG00000027179* | *SIM2* | 13: 200,355,606-200,403,247 | embryonic pattern specification |

Genes that located within the differential methylation regions or closest to the differential methylation regions of the intergenic region were defined as DMGs to perform gene function enrichment analysis via Gene Ontology (GO).
