# Supplementary material for: Whole-Genome Methylation Analysis Reveals Epigenetic Variation in Cloned and Donor Pigs
Source: Front Genet. 2020 Feb 20;11:23. doi: 10.3389/fgene.2020.00023 (PMC7046149; doi:10.3389/fgene.2020.00023)
Supplement: Supplementary file 1 [file DataSheet_1.zip › Sup Material/Sup File S9.DOCX]

# Supplementary File 9

DMGs enriched to reproduction related pathways in the blood

| Gene ID | Gene name | DMG Location | KEGG pathway |
| --- | --- | --- | --- |
| *ENSSSCG00000000555* | *ITPR2* | 5:46,935,923-47,464,125 | Oocyte meiosis, Oxytocin signaling pathway , Estrogen signaling pathway, GnRH signaling pathway |
| *ENSSSCG00000011141* | *CALML5* | 10:65406633-65407082:1 | Oocyte meiosis, Oxytocin signaling pathway , Estrogen signaling pathway, GnRH signaling pathway |
| *ENSSSCG00000035088* | *novel gene* | 8:119,587,111-119,739,310 | Oocyte meiosis, Oxytocin signaling pathway |
| *ENSSSCG00000007520* | *GNAS* | 17: 58,998,981-59,055,340 | Ovarian steroidogenesis, Oxytocin signaling pathway, Estrogen signaling pathway, GnRH signaling pathway |
| *ENSSSCG00000012050* | *RCAN1* | 13:198,106,257-198,205,629 | Oxytocin signaling pathway |
| *ENSSSCG00000017206* | *GRB2* | 12: 5,940,645-6,010,836 | Prolactin signaling pathway, Estrogen signaling pathway, GnRH signaling pathway |
| *ENSSSCG00000031366* | *novel gene* | 8: 98,082,496-98,082,714 | Oocyte meiosis |

Genes that located within the differential methylation regions or closest to the differential methylation regions of the intergenic region were defined as DMGs to perform KEGG pathway enrichment analysis.
