# Supplementary material for: Whole-Genome Methylation Analysis Reveals Epigenetic Variation in Cloned and Donor Pigs
Source: Front Genet. 2020 Feb 20;11:23. doi: 10.3389/fgene.2020.00023 (PMC7046149; doi:10.3389/fgene.2020.00023)
Supplement: Supplementary file 1 [file DataSheet_1.zip › Sup Material/Image S1.DOC]

# Supplementary figure 1

**The chromosomal methylation of the samples**

(A), (B), (C), (D) is the chromosomal methylation of DP-blood, CP-blood, DP-ear and CP-ear, respectively. The circle colors indicates the type of methylation, and the height of histogram represents the proportion of the methylation.
